# Fibrinogen metabolic responses to trauma

**DOI:** 10.1186/1757-7241-17-2

**Published:** 2009-01-13

**Authors:** Wenjun Zhou Martini

**Affiliations:** 1 U.S. Army Institute of Surgical Research, Ft. Sam Houston, TX 78234, USA

## Abstract

Coagulation complications are significant contributors to morbidity and mortality in trauma patients. Although the lethal triad of hypothermia, acidosis and coagulopathy has been recognized for over a decade, the underlying mechanisms related to the development of coagulopathy remain unclear. Recent data suggest that decreased fibrinogen levels contribute to the development of coagulation disorders. Thus, regulation of fibrinogen availability, not fully understood at present, may play an important role in survival of trauma patients. This review summarizes the recent findings of the studies that have explored mechanisms related to changes in fibrinogen availability following trauma-related events. Trauma alters fibrinogen metabolism in a variety of ways: hemorrhage – accelerated fibrinogen breakdown; hypothermia – inhibited fibrinogen synthesis; and, acidosis – accelerated fibrinogen breakdown. However, hemorrhage, hypothermia andcidosis all result in a consistent outcome of fibrinogen availability deficit, supporting the notion of fibrinogen supplementation in trauma patients with coagulation defects. Future prospective clinical trials are needed to confirm the beneficial effects of fibrinogen supplementation in trauma patients with bleeding complications.

## Introduction

Coagulation complications are significant contributors to morbidity and mortality in trauma patients [1,2]. Mortality in patients with severe injuries and coagulopathy is found to be four times greater than in patients with injuries alone [3]. Trauma-related coagulopathy is associated with hypoperfusion due to tissue injury and blood loss, hemodilution from resuscitation with crystalloid and/or colloid solutions, progressive hypothermia and the development of acidosis. Since the recognition of the lethal triad of hypothermia, acidosis, and coagulopathy over a decade ago [1,2], a great deal of effort has been made to elucidate possible mechanisms contributing to trauma related coagulopathy as well as to search for effective treatments [4-7]. Recent data suggest that fibrinogen availability may play an important role in the survival of patients. The purpose of this article is to review recent findings that have been made concerning clotting protein fibrinogen metabolism and availability following trauma-related events, including hemorrhage, resuscitation, hypothermia and acidosis.

### Fibrinogen Availability

As the precursor of clot formation, fibrinogen plays an important role in coagulation function. Fibrinogen deficiency is associated with uncontrolled bleeding and compromised survival [8-12]. Thus, regulation of fibrinogen availability is critical to survival in trauma patients.

As an acute phase protein, fibrinogen is synthesized in the liver and released into the circulation. It is catabolized through normal protein degradation, the coagulation process, and other unknown pathways. At any moment, fibrinogen availability is delineated by the dynamic balance of synthesis and breakdown. Mathematically, fibrinogen availability can be expressed as:

Fibrinogen availability = [[Fibrinogen] × total plasma volume] + [synthesis rate × time] - [breakdown rate × time]

Where [fibrinogen] is the initial fibrinogen concentration (mg/dL); synthesis is the amount of fibrinogen (mg) produced in a unit of time (hour); and breakdown rate is the amount of fibrinogen (mg) consumed in a unit of time (hour).

The importance of initial fibrinogen concentration is demonstrated by its relationship to maximum allowable blood loss before casualties reach critical status. Using a mathematical model to analyze hemostasis during blood loss that was validated with clinical data collected from 208 consecutive patients, Singbartl et al. showed that the maximal allowable blood loss is highly dependent on the initial fibrinogen levels [13]. For instance, for a given hematocrit (e.g., 45%) and platelet count (e.g., 225 × 10^3^/μL), when three representative initial fibrinogen levels are 450 mg/dL, 300 mg/dL, and 200 mg/dL, the respective maximum allowable blood losses are 3750 ml, 1900 ml, and 750 ml, before critical levels of fibrinogen are reached. Although the exact critical levels of fibrinogen at which bleeding complications are provoked are debatable, the point is that bleeding complications do not always happen after large amounts of blood loss and hemodilution. Patients with low initial fibrinogen levels may possibly develop bleeding complications following even moderate blood loss and hemodilution. These patients may also have other pathological conditions present that could contribute to the development of coagulopathy [4,5,14-16].

Fibrinogen concentration is routinely measured by clinical laboratories. In contrast, quantification of fibrinogen synthesis and breakdown presents technical challenges. Consequently, fibrinogen concentration has been the only measurement available to assess changes in fibrinogen. It is worth clarifying that fibrinogen concentration does not in and of itself reveal any information about synthesis or breakdown. For instance, an increased fibrinogen concentration may be due to decreased consumption, and/or increased production, or simply deceases in plasma volume. Thus, to fully understand changes in fibrinogen availability requires information of fibrinogen metabolism.

### Quantification of Fibrinogen Metabolism

The important role of fibrinogen in coagulation led to investigations to study fibrinogen kinetics. In the 1960s and 1970s, fibrinogen synthesis was quantified using radioactive isotope labeled amino acids in experimental animals [17-19]. Upon administration, radioactive labeled amino acids were incorporated into fibrinogen molecules and the increased radioactivities in fibrinogen over time were used to reflect fibrinogen production. By comparing the radioactivities in fibrinogen with the control group, this approach allowed assessment of changes in fibrinogen production, but did not offer any information about fibrinogen breakdown. To measure fibrinogen catabolism, others used radioactive labeled fibrinogen to investigate fibrinogen breakdown in experimental animals and humans [20-25]. In this approach, radioactive labeled fibrinogen (i.e., ^125^I – fibrinogen) was injected into subjects and blood samples were withdrawn daily for 5 to 14 days afterwards. Fibrinogen was then isolated from the blood samples and its radioactivities were measured. The decreases of radioactivities of fibrinogen over time were used to reflect fibrinogen breakdown. This approach was used in the past as an *in vivo *means to assess changes of fibrinogen breakdown under different pathophysiological states [20-25]. Unfortunately, this approach did not allow quantification of fibrinogen synthesis. Consequently, changes in fibrinogen metabolism remained unclear due to the lack of a methodology to quantify fibrinogen synthesis and breakdown.

Recently, Martini et al. developed an *in vivo *methodology to quantify fibrinogen synthesis and breakdown rates simultaneously and independently [26]. The simultaneous and independent quantification of synthesis and breakdown provides comprehensive and complete assessment of fibrinogen metabolism and availability. This methodology involves the infusion of stable isotope labeled amino acids with subsequent gas chromatography mass spectrometry analysis. With the infusion of differently labeled amino acids for different durations, the incorporation of the isotopic labels in fibrinogen over time is used to calculate fibrinogen synthesis, while the decay of the isotopic labels in fibrinogen after the infusion ceases is used to calculate fibrinogen breakdown. There are several advantages to this methodology. First, stable isotopes are naturally occurring and safe for use in humans; second, synthesis and breakdown rates are quantified simultaneously and independently in the same subject; and third, the entire study (infusion and blood samplings) takes only 6 to 8 hours, compared to the days required in previous approaches. The establishment of this methodology made it possible to investigate changes of fibrinogen metabolism in trauma.

### Effects of Hemorrhage

Hemorrhage is the leading potentially preventable cause of death on the battlefield and a major cause of death in civilian trauma [11,27]. One of the most detrimental complications following hemorrhage is the disruption of hemostasis, resulting in uncontrolled bleeding, disseminated intravascular coagulation (DIC), and thrombotic complications [8,10-12]. Based on the limited data available at present, changes in fibrinogen availability are involved in the development of clotting disorders. In acutely injured trauma victims, fibrinogen levels were observed to be the first coagulation proteins and factors to drop to pathophysiological levels [8,9], although this is not a universal finding [28]. The observed decrease of fibrinogen was not found to be attributable to blood loss or resuscitation [8,9].

To investigate the effects of hemorrhage on fibrinogen metabolism, a swine model was used in which a moderate hemorrhage was induced by withdrawing 35% of estimated total blood volume from the femoral artery [26]. After hemorrhage and stabilization, a stable isotope infusion was performed with 1-^13^C- phenylalanine for 6 hours and d_5_-phenylalanine for 4 hours. During the infusion, blood samples were collected hourly and isotopic enrichments of fibrinogen from the infusion were determined using gas chromatography mass spectrometry analysis. Data from this study showed that after moderate hemorrhage, fibrinogen breakdown was accelerated compared with the control group. There was no change in the fibrinogen synthesis rate (Additional file 1) [26]. The deficit between fibrinogen production and consumption indicates a potential decrease in fibrinogen availability after hemorrhage. It is worth mentioning that there were no changes in pH or temperature associated with the 35% blood loss in this study, suggesting that the effects observed in the study were due to hemorrhage only. Under more severe hemorrhagic shock, changes of fibrinogen metabolism may be different due to possible changes in pH or temperature resulted from severe blood loss and compromised tissue perfusion.

### Effects of lactated Ringer's Resuscitation

To treat hemorrhagic shock, fluid resuscitation is routinely used in clinical practice to restore tissue perfusion. The effects of various crystalloids and colloids with different volume-expanding capacities have been reported in the literature [29-33]. Among these fluids, lactated Ringers solution (LR) is considered part of standard care [34]. The effects of LR on fibrinogen kinetics were reported recently by Martini et al. in a study of swine [35]. In the study, a moderate hemorrhage was induced by withdrawing 35% of estimated blood volume in 12 pigs, with an additional 6 pigs used as controls. Afterwards, the 12 hemorrhaged pigs were randomly divided into the hemorrhage only group and hemorrhage-LR resuscitation group. In the hemorrhage-LR resuscitation group, LR solution at 3 times the bled volume was given to the pigs while no fluid was given in the hemorrhage only group. Upon stabilization, stable isotope infusion was performed in all three groups, followed by blood drawn hourly and subsequent gas chromatography mass spectrometry analysis. Data from this study [35] showed that compared with the control value (3.0 ± 0.5 mg/kg/h), fibrinogen breakdown was similarly increased in both the hemorrhage only group (5.4 ± 0.7 mg/kg/h) and the hemorrhage-LR resuscitation group (5.6 ± 0.5 mg/kg/h). There were no significant differences in fibrinogen synthesis among the control group (2.5 ± 0.6 mg/kg/h), the hemorrhage only group (1.7 ± 0.3 mg/kg/h) or the hemorrhage-LR resuscitation group (2.6 ± 0.4 mg/kg/h, Additional file 1). This suggests that the changes in fibrinogen metabolism resulted from hemorrhagic shock and that LR resuscitation itself did not affect fibrinogen metabolism. However, the effects of other resuscitation fluids, such as colloids, on fibrinogen metabolism remain to be investigated.

### Effects of Hypothermia

Hypothermia, clinically defined as a body temperature of 34°C or less, is commonly observed in severely injured trauma patients [36]. The association of hypothermia to coagulation dysfunction and mortality has been well described [36-41]. Compared with patients who had a body temperature of 36.1 ± 0.7°C, there was a 2.4-fold increase in blood loss in post-laparotomy patients whose body temperature was 33.8 ± 0.5°C [37]. In a group of trauma patients with Injury Severity Scores (ISS) greater than 25, the mortality increased from 10% to 100% when body temperature declined from 35°C to less than 32°C [40]. Among nonsurviving trauma patients, approximately 80% have body temperatures of less than 34°C at the time of death [38]. The adverse effects of hypothermia on coagulation have been indicated by prolonged prothrombin time (PT) and activated partial thromboplastin time (aPTT) in hypothermic patients and experimental animals, as well as in plasma samples cooled in vitro [42-47]. The dynamic changes of fibrinogen metabolism during hypothermia were revealed recently by Martini et al [48].

In a normovolemic swine model, hypothermia of 32°C was induced using a cold blanket with circulating water at 4°C [48]. Temperature of 32°C was used based on the fact that 100% mortality was observed when the temperature in trauma patients dropped below 32°C [40]. When the animal temperature was lowered to 32°C and stabilized, stable isotope 1-^13^C-phenylalanine was infused for 6 hours and d_5_-phenylalanine was infused for 4 hours. Blood samples were taken hourly during the infusion and the isotopic labeling of fibrinogen was determined using gas chromatography and mass spectrometry analysis. It was found that hypothermia decreased fibrinogen synthesis, with no effects on fibrinogen breakdown (Additional file 1) [48]. This observation indicates that, in response to cooling, fibrinogen synthesis and degradation are regulated via different mechanisms and that there is a potential deficit in fibrinogen availability following hypothermia. The metabolic changes in fibrinogen were associated with prolonged clotting initiation time and decreased clotting speed.

### Effects of Acidosis

Among all the factors contributing to coagulation disorders, acidosis is one of the most important predictors of coagulopathy in trauma patients [49], with the likelihood of death increasing as the severity of acidosis increases [1,38,50-52]. The detrimental effects of acidosis on coagulation include impaired enzyme activities, depleted fibrinogen levels and platelet counts, prolonged clotting times, and increased bleeding times [1,38,50-53]. The mechanisms contributing to the depletions of fibrinogen were reported recently by Martini et al [48].

In a swine model, acidosis of pH 7.1 was induced by an infusion of 0.2 N HCl in LR [54]. When the target pH of 7.1 was achieved and stabilized, a stable isotope infusion of 1-^13^C-phenylalanine and d_5_-phenylalanine was performed with hourly blood sampling. Following gas chromatograph and mass spectrometry analysis, data from this study showed that, in contrast to the effects of hypothermia, acidosis increased fibrinogen breakdown by 1.8-fold compared with control values, with no effects on fibrinogen synthesis (Additional file 1) [54]. Thus, it appears that there were differential effects on fibrinogen synthesis and breakdown by acidosis and there was a potential depletion of fibrinogen availability following acidosis.

### Fibrinogen Supplementation

Despite the differential effects on fibrinogen synthesis and breakdown, hemorrhage, hypothermia and acidosis all resulted in a single outcome: a deficit in fibrinogen availability. Beneficial effects of fibrinogen supplementation were reported in animal and *in vitro *studies following the administration of Haemocomplettan P^®^, a fibrinogen concentrate available in European countries [55-60] (some of these studies were conducted or sponsored by the manufacture). In pigs with 60% blood volume exchanged with hydroxyethyl starch, Fries et al. investigated the effects of Haemocomplettan P^® ^together with prothrombin complex concentrate on blood loss from a standard liver laceration and survival [55]. The blood loss in the supplement group was 240 ml (50 – 830 ml) compared to 1800 ml (1500 – 2500 ml) in the placebo group. All animals in the supplement group survived compared to 20% survival in the placebo group [55]. Similarly, in a rat model with sepsis-induced DIC, Kaspereit et al. reported significant decrease in mortality following treatment with Haemocomplettan P^® ^[58]. Thus, fibrinogen supplementation may be potentially beneficial following massive blood loss and DIC. It remains unclear, however, how early fibrinogen supplementation affects the dynamic features of fibrinogen metabolism.

Consistent with animal studies, decreased fibrinogen levels have been documented in coagulopathic patients [61-63]. In search for effective treatments, efforts have been made to evaluate outcomes from transfusion of blood products, such as fresh whole blood, fresh frozen plasma (FFP), cryoprecipitate, and red blood cells (RBC). Recent published retrospective studies suggested potential benefits of transfusion of a higher ratio of fibrinogen to red blood cell unit ratio in trauma patients [6,7,64-72].

To observe the effects of fibrinogen supplementation on survival, Stinger et al. performed a retrospective study in massively transfused trauma patients at a US Army combat support hospital [72]. Two hundred fifty-two trauma patients with an average ISS of 21 ± 10 who received 10 or more units of RBC in 24 hours were included in the study. The amount of fibrinogen transfused was calculated based on fibrinogen amount within each blood product, such as fresh whole blood, cryoprecipitate, aphaeresis platelets and FFP. The ratio of fibrinogen-to-RBC was used to identify two patient groups: a low ratio group (ratio < 0.2 g fibrinogen per RBC unit) and a high ratio group (ratio ≥0.2 g fibrinogen per RBC unit). The authors reported mortality rates of 52% and 24% in the low and high ratio groups, respectively (p < 0.001). Upon logistic regression analysis, the fibrinogen-to-RBC ratio was found to be independently associated with mortality [72]. It should be mentioned, however, that some recent studies have called into question the value of high fibrinogen-to-RBC ratio in treatment of trauma patients [73,74]. Future prospective clinical trials are needed to clarify the effects of a higher ratio of fibrinogen to RBC transfusion and outcome in trauma patients.

## Conclusion

Fibrinogen availability is regulated through synthesis and breakdown to maintain coagulation function. Recent studies have revealed the mechanisms underlying changes in fibrinogen availability following trauma. Hemorrhage, hypothermia and acidosis alter fibrinogen metabolism in a variety of ways: First, moderate hemorrhage accelerated fibrinogen breakdown with no significant effects on fibrinogen. Second, hypothermia of 32°C inhibited fibrinogen synthesis with no effects on fibrinogen breakdown. Finally, acidosis of pH 7.1 accelerated fibrinogen breakdown without changing fibrinogen synthesis. Despite the differential effects on fibrinogen synthesis and breakdown, hemorrhage, hypothermia and acidosis all lead to a deficit in fibrinogen availability. Recent retrospective clinical studies in trauma patients and animal trials suggest that fibrinogen supplementation may be beneficial. Further prospective clinical trials to confirm the benefits of fibrinogen supplementation in trauma patients are warranted.

## Abbreviations

DIC: disseminated intravascular coagulation; LR: lactated Ringer's solution; ISS: Injury Severity Scores; PT: prothrombin time; aPTT: activated partial thromboplastin time; FFP: fresh frozen plasma; RBC: red blood cells.

## Competing interests

The author declares that the author has no competing interests.

## Authors' contributions

The author drafted the manuscript.

## Supplementary Material

Additional file 1 **The effects of hemorrhage, LR resuscitation, hypothermia, and acidosis on fibrinogen synthesis and breakdown in pigs**. Data presented were collected during studies conducted by Martini et al [26,35,48,54]. *p < 0.05 compare with control values. Click here for file
