# Supplementary figures and images for: Fibrinogen metabolic responses to trauma
Source: Scand J Trauma Resusc Emerg Med. 2009 Jan 13;17:2. doi: 10.1186/1757-7241-17-2 (PMC2667162; doi:10.1186/1757-7241-17-2)

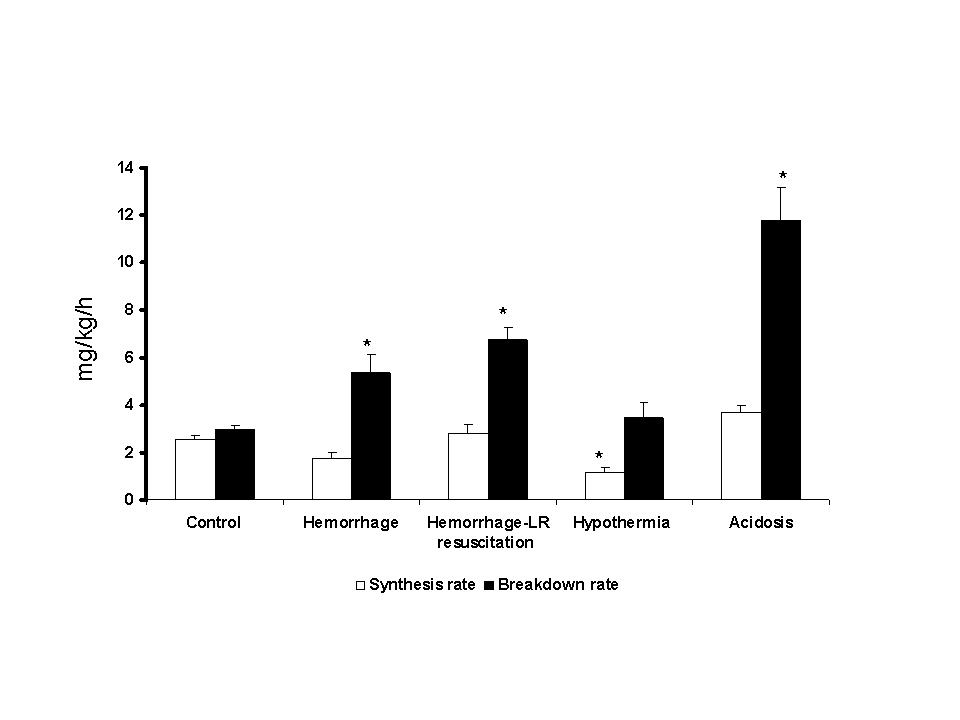

Supplement: Additional file 1 — The effects of hemorrhage, LR resuscitation, hypothermia, and acidosis on fibrinogen synthesis and breakdown in pigs. Data presented were collected during studies conducted by Martini et al [26,35,48,54]. *p < 0.05 compare with control values. [file 1757-7241-17-2-S1.jpeg]
